# Microwave Synthesis, Characterization, and Photoluminescence Properties of Nanocrystalline Zirconia

**DOI:** 10.1155/2014/349457

**Published:** 2014-01-21

**Authors:** A. K. Singh, Umesh T. Nakate

**Affiliations:** ^1^DRDO Center for Piezoceramics and Devices, ARDE, Pashan, Pune 411021, India; ^2^Nanomaterials and Sensors Laboratory, Defence Institute of Advanced Technology (DU), Girinagar, Pune 411025, India

## Abstract

We report synthesis of ZrO_2_ nanoparticles (NPs) using microwave assisted chemical method at 80°C temperature. Synthesized ZrO_2_ NPs were calcinated at 400°C under air atmosphere and characterized using FTIR, XRD, SEM, TEM, BET, and EDS for their formation, structure, morphology, size, and elemental composition. XRD results revealed the formation of mixed phase monoclinic and tetragonal ZrO_2_ phases having crystallite size of the order 8.8 nm from most intense XRD peak as obtained using Scherrer formula. Electron microscope analysis shows that the NPs were less than 10 nm and highly uniform in size having spherical morphology. BET surface area of ZrO_2_ NPs was found to be 65.85 m^2^/g with corresponding particle size of 16 nm. The band gap of synthesized NPs was found to be 2.49 eV and PL spectra of ZrO_2_ synthesized NPs showed strong peak at 414 nm, which corresponds to near band edge emission (UV emission) and a relatively weak peak at 475 and 562 nm.

## 1. Introduction

ZrO_2_ (zirconia) is a material of great technological importance, having good natural color, high strength, high toughness, high chemical stability, excellent corrosion, and chemical and microbial resistance [1, 2]. ZrO_2_ is a wide band gap P-type semiconductor that exhibits abundant oxygen vacancies on its surface. The high ion exchange capacity and redox activities make it useful in many catalytic processes as a catalyst [3]. ZrO_2_ is also an important dielectric material that is being investigated for potential application as an insulator in transistors in future nanoelectric devices [4]. Garcia et al. [5] have highlighted its potential to replace SiO_2_ in advanced metal oxide semiconductor (MOS) devices and in optical applications.

ZrO_2_ has three well-defined crystal structures/phases, that is, cubic (c-ZrO_2_), tetragonal (t-ZrO_2_), and monoclinic (m-ZrO_2_), under normal atmosphere and at different temperatures [6, 7]. Generally, m-ZrO_2_ phase is thermodynamically stable up to 1100°C, t-ZrO_2_ phase exists in the temperature range of 1100–2370°C, and the cubic phase is found above 2370°C [8]. There are contradictory reports on the existence of various phases under different conditions of temperatures. Recently, existence of t-ZrO_2_ at low temperature has been reported by many researchers [9–11]. Ciuparu et al. [9] have reported the initial existence of amorphous phase, and after heating above 750°C, t-phase formation has been observed, and transition from tetragonal to monoclinic has been observed after calcinations at 1200°C. Shukla et al. [11] have observed nucleation of t-ZrO_2_ phase from the amorphous phase at 400°C, which becomes stable at 600°C, and after calcination at 800°C complete tetragonal-to-monoclinic phase transformation has been reported.

ZrO_2_ has many interesting characteristics, such as superior mechanical characteristics, increased fracture toughness, hardness, strength, high thermal expansion coefficient, low thermal conductivity, superplastic deformation, phase stability, and good chemical resistance, which have resulted in a variety of industrial and engineering applications that include high durability coating, catalytic agents, medical prosthetics, cutting tools, synthetic jewels, high density grinding media, wear components, bearings, seals, valves, nose cones and tiles for space shuttles and missiles, turbines, and the most demanding aviation engines [12–17]. It also has the best performance as ceramic dental material. ZrO_2_ has found uses in solid oxide fuel cells [18] and in NO_x_, O_2_ gas sensors [19]. The fully stabilized ZrO_2_ is also well suited for high temperature energy conversion systems, due to its high oxygen ion transport capabilities and good long-term stability.

In spite of many unique characteristics, it has serious drawback for engineering application because it undergoes phase transformation with a change in volume at high temperatures, whereas, for optical and electronic applications, precise evaluation of its band gap is of significance since wide variation has been reported in its band gap [5, 9]. ZrO_2_ is usually in the form of m-ZrO_2_ at room temperature. Thus, the synthesis and room temperature stabilization methods of nano-t-ZrO_2_ are very important and of scientific significance with application prospects.

To prepare nano-ZrO_2_, several methods have been reported that include sol-gel, flame spray, combustion, glycothermal process, hydrothermal processing, and precipitation routes [7, 20–24]. In particular, a chemical solution method, which involves formation of a stable particle by a direct reaction between the atoms or reaction species, provides the most suitable way of synthesizing a sample of imperfection/defect free particles. The sol-gel method is one of the most common methods that have been used to synthesize nano-ZrO_2_. Many of these methods of nano-ZrO_2_ preparation use a synthetic template material, which is not economical and relatively more expensive. In the literature, there are few reports on the usage of microwave method for the synthesis of ZrO_2_ [10, 25, 26] which is the novel route of synthesis of metal oxide NPs, being clean, cost-effective, energy efficient, eco-friendly, rapid and convenient method of heating, and results in higher yields in shorter reaction times [27]. Here, we report simple, low temperatures of synthesis of nanocrystalline ZrO_2_ NPs using microwave assisted method. To the best of our knowledge, the reaction temperature we used is the lowest used in the synthesis of ZrO_2_ NPs. The synthesized NPs of ZrO_2_ were investigated using FTIR, XRD, SEM-EDS, and TEM analysis.

## 2. Experimental Details

### 2.1. Synthesis

All chemicals used in the synthesis have been used as received from chemical suppliers without any further purification and processing. 50 mL of 0.1 M zirconium oxychloride octahydrate solution was prepared and 15 mL 1 N NaOH solution was added dropwise in given solution with continuous stirring, after precipitation precursor solution was kept in microwave oven for 12 min with power 420 W and temperature 80°C using RAGA'S microwave system. Microwave used for this experiment was of power range of 140 W to 700 W. Obtained precipitate was filtered, washed 2-3 times with DI water (18.2 MΩ·cm resistivity) and then dried at temperature 150°C for several hours. Dried powder was crushed and calcinated in air atmosphere at 400°C for 2 hours and obtained sample was characterized.

### 2.2. Characterization

The prepared annealed samples were characterized for their formation, structure, morphology, and elemental composition using Fourier transform infrared (FTIR) spectroscopy, X-ray diffraction analysis (XRD), scanning electron micrograph (SEM), transmission electron micrograph (TEM), energy dispersive spectrometer (EDS), Brunauer, Emmett and Teller (BET), and UV-Vis spectroscopy. FTIR of samples was performed using Shimadzu Affinity-1 FTIR spectrometer. Crystallographic study was carried out using Bruker AXS, Germany (Model D8 Advanced), diffractometer in the scanning range of 20–80° (2*θ*) using Cu K*α* radiations of wavelength 1.5406 Å. JEOL ASM 6360A scanning electron microscope (SEM) was used to study the morphology and the elemental analysis. Transmission electron microscopy (TEM) of samples was carried out using FEI-Tecnai G^2^20. Measurement of BET surface area was carried out for nitrogen adsorption using a Micromeritics Ins., USA. UV-Vis spectroscopy of samples was done in the range of about 200–800 nm with the help of Ocean Optics HR4000 high-resolution spectrometer. Room temperature photoluminescence properties of NPs were studied using Perkin Elmer LS55 Luminescence Spectrometer in spectral range of 300–800 nm in the wavelength range of 270–320 nm as excitation source.

## 3. Results and Discussion

### 3.1. FTIR Analysis

**Figure 1:**
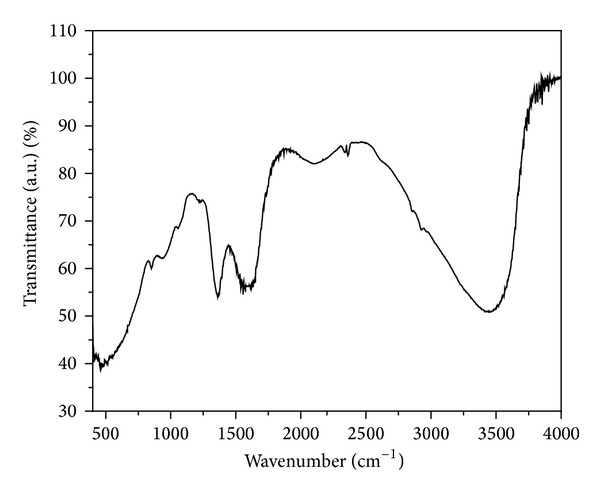
FTIR spectra of ZrO_2_ of NPs in the 400–4000 cm^−1^ region.

In order to ascertain the molecular nature of the synthesized material, the FTIR spectrum of the ZrO_2_ sample was taken as shown in Figure 1. The spectrum of ZrO_2_ depends on the nature of the material, preparative procedures used, solid-state structure, and so forth. The observed strong FTIR absorption peak at about 470 cm^−1^ region is due to the Zr–O vibration, which confirm the formation of ZrO_2_ structure [28], prominent peak of 1380 cm^−1^ region corresponds to O–H bonding, peak in the region of 1553 cm^−1^ may be due to the adsorbed moisture and in the 3425–3495 cm^−1^ region is attributed to stretching of O–H groups, characteristic of a highly hydrated compound [28].

### 3.2. Crystallographic Analysis

**Figure 2:**
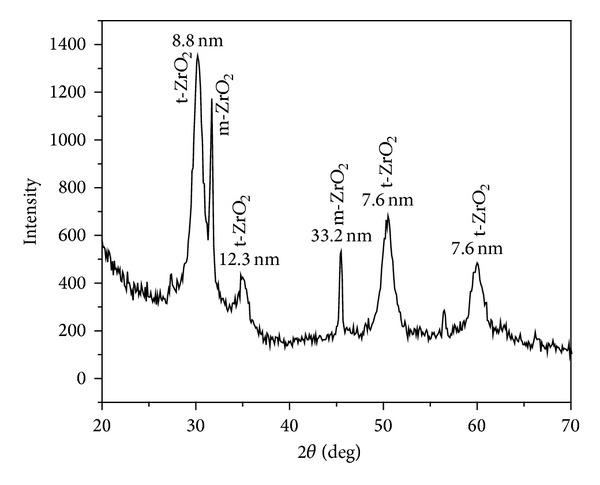
XRD pattern of ZrO_2_ NPs calcinated at 400°C.

To confirm the phase formation of ZrO_2_, XRD pattern of the sample was recorded after calcination in air atmosphere and is shown in Figure 2. The narrow line widths indicate high crystalline nature of the synthesized material. As reported by Shukla et al., [29] scan range of 27–32 degrees contained the strongest lines for monoclinic as well as tetragonal phases. Observed XRD peaks (Figure 2) for sample calcinated at 400°C indicate the formation of t-ZrO_2_ and m-ZrO_2_ mixed phases. Selection of calcination of sample at 400°C was based on earlier reports indicating that, in order to crystallize ZrO_2_, OH ion must be removed under thermal treatment and ZrO_2_ is crystallized at a temperature of about 400°C [23]. The distinguishing characteristic peaks for tetragonal occur at 2*θ* = 30.2, 34.5, 50.2, and 60.2 corresponding to the (101), (110), (200), and (211) reflections [JCPDS No. 70-1769] [30, 31]. Stabilization of t-phase at lower temperature is most likely due to low surface energy of the t-phase relative to m-phase. At 300°C, zirconia has an amorphous structure and pure ZrO_2_ is crystallized at a temperature of about 400°C [23], but in our case we have observed mixed phase formation.

Mixed phase formation of ZrO_2_ is a common feature in zirconia synthesis and has been reported by many authors [28]. Tyagi et al. [23] have reported t-ZrO_2_ phase formation at 400°C, and with increase in calcinations temperature they have reported m and t-ZrO_2_. The occurrence of metastable tetragonal phase is attributed to the critical crystallite size effect as reported by Garvie [32]. Garvie experimentally showed the existence of a critical size of ~30 nm, below which the metastable t-phase is stable and above which the m-phase is stable.

The crystallite size of NPs has been calculated by XRD line broadening of peak using Scherrer's formula [33]

(1) $$\begin{array}{c} D=\frac{K\lambda }{\beta \mathrm{Cos}\theta }, \end{array}$$

where *K* is 0.9, *λ* is wave length of X-ray source (0.1540598 nm), *β* is full width at half maximum in radians, and *θ* is Bragg's diffraction angle. Crystallite sizes, calculated using Scherrer's formula (1), are indicated in Figure 2 corresponding to each peak and are the order of 8.8 nm from most intense XRD peak, whereas average size is about 14 nm which is in close agreement with the particle size of ZrO_2_ as obtained from BET surface area analysis as discussed.

### 3.3. Elemental and Morphological Analysis

**Figure 3:**
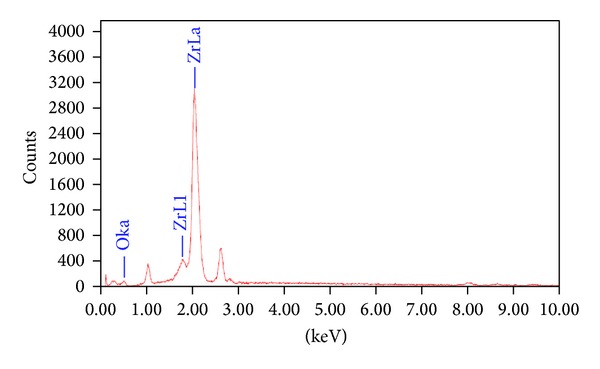
EDAX spectra of ZrO_2_ of NPs calcinated at 400°C.

The synthesis of ZrO_2_ NPs was confirmed by recording EDAX spectra and is depicted in Figure 3. Emission peaks, such as OKa, ZrL1, and ZrLa were observed in the EDAX spectrum confirmed the stoichiometry of synthesized NPs. It shows that Zr and O elements are present almost in stiochoimetric ratio.

**Figure 4:**
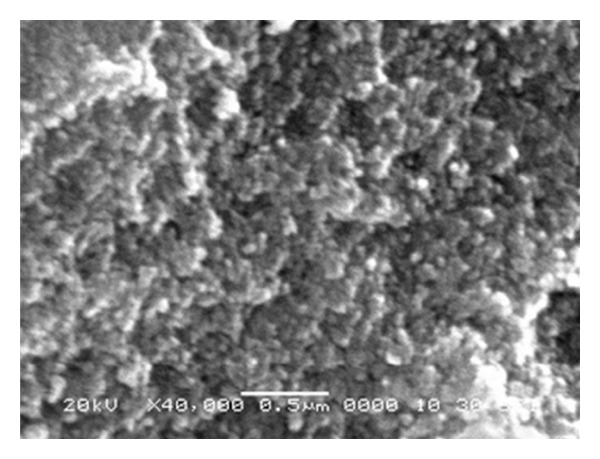
SEM micrographs of ZrO_2_ NPs.

**Figure 5:**
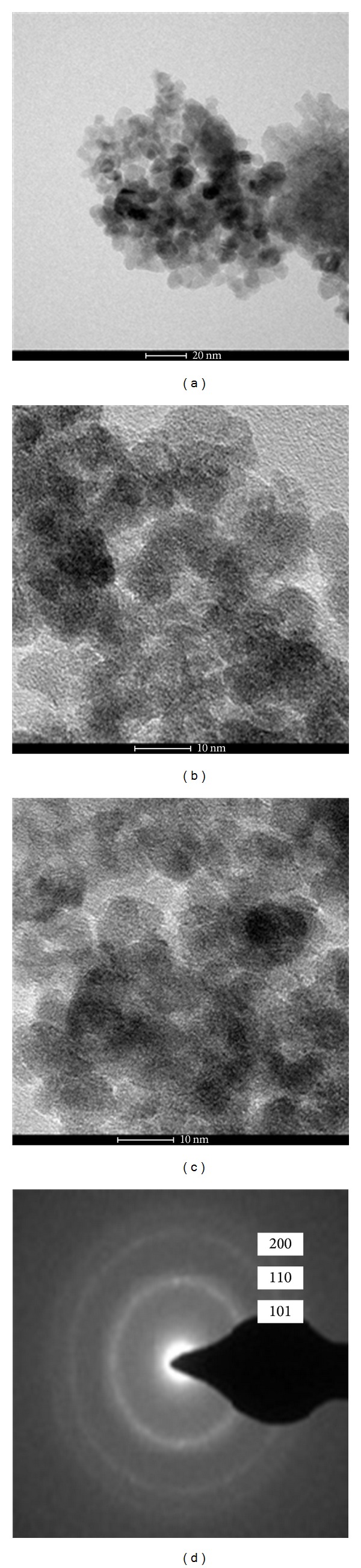
TEM micrographs of ZrO_2_ NPs at different magnifications (a)–(c) and electron diffraction pattern taken on a selected area of the sample (d).

Morphological investigations of 400°C air calcinated ZrO_2_ sample were carried out using SEM and TEM and are shown in Figures 4 and 5, respectively. The morphological characterization highlighted the importance of nanocrystalline ZrO_2_ preparation in maintaining the nanostructured phase. It is clear from Figure 4 that NPs are of uniform size. Observation of Figure 4 shows that ZrO_2_ particles are spherical in nature and size of the particles is in the nm regime, but size could not be finely resolved from SEM. For the purpose, TEM of sample has been shown in Figure 5. As can be seen from TEM micrographs of sample, some agglomeration has been observed due to different t- and m- phases present in the sample. In spite of agglomeration of the NPs, it can be observed that the sizes of the particles are of the order 10 nm. The TEM results are also supporting the results of other analyses based on XRD and BET surface area calculations.

Figure 5(d) shows an electron diffraction pattern indicating well-defined quasicontinuous diffraction rings of the sample that calcined at 400°C for 2 h. It is noticeable that the (101), (110), and (200) planes are clearly distinguished [34] as observed in XRD patterns.

### 3.4. BET Analysis

Surface area analysis was done by nitrogen absorption using BET surface analyzer. NPs size has been calculated from surface area assuming NPs to be of spherical shape, using the following equation [35]:

(2) $$\begin{array}{c} \;\;d=\frac{6}{\rho S_{\text{BET}}}, \end{array}$$

where *ρ* is the density of ZrO_2_ particles and *S*_BET_ is the BET surface area for powder sample calcinated at 400°C; measured BET surface area has been found to be 65.85 m^2^/g. Particle size of ZrO_2_ NPs was found to be 16 nm from (2) which is in agreement with the above discussion under the assumption used in the analysis.

### 3.5. Band Gap Analysis

**Figure 6:**
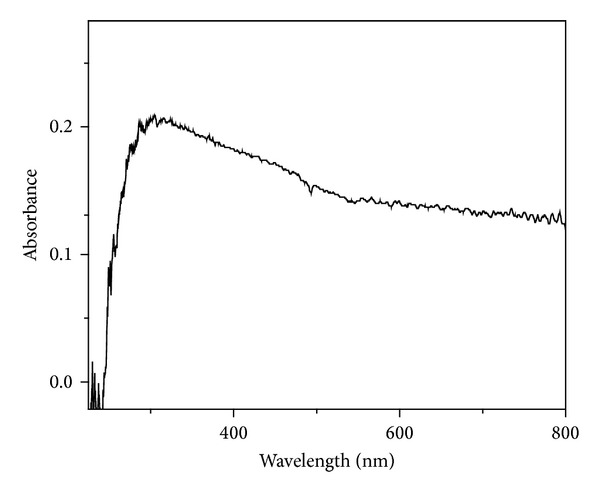
UV-Vis absorbance spectra of microwave synthesized ZrO_2_ sample calcinated at 400°C.

**Figure 7:**
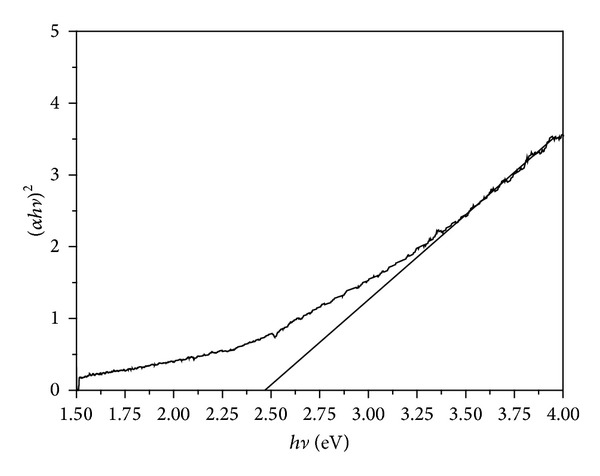
Plot of (*α* *hν*)^2^ versus *hν* for ZrO_2_ sample calcinated at 400°C.

**Figure 8:**
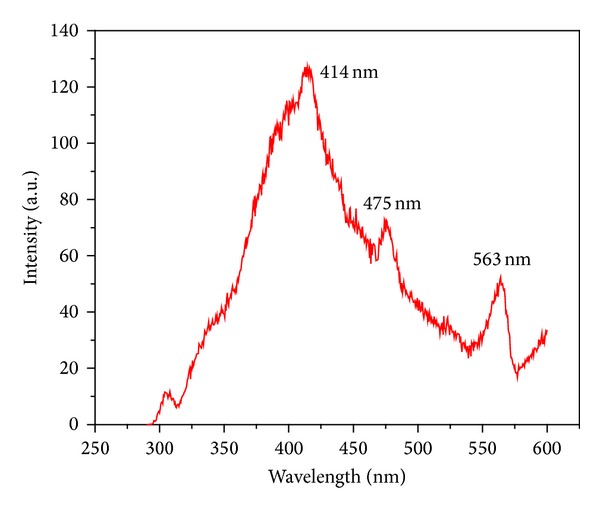
PL spectra of microwave synthesized ZrO_2_ sample excited at wavelength 270 nm.

Reduction in the nanoparticle size can cause change in the optical band gap of metal oxides through the narrowing of the valence and conduction bands [5]. The other important factors that can affect optical band gap are defect centers, mechanical stress, and changes in the crystallinity. Optical absorption spectra of NPs, as shown in Figure 6, have been studied without taking into account the reflection and transmission losses. Tauc's relation of the absorption coefficient (*α*) with the photon energy (*hν*) has been used to determine the band gap energy of sample and is given by

(3) $$\begin{array}{c} \alpha =\frac{\alpha_{0}\left(h\nu -E_{g}\right)}{h\nu }, \end{array}$$

where *α* is the absorption coefficient, *α*_0_ is the constant, *hν* is the photon energy, and *E*_*g*_ is the band gap energy of the material. The value of *n* depends on the probability of transition; it takes values as 1/2, 3/2, 2, and 3 for direct allowed, direct forbidden, indirect allowed, and indirect forbidden, respectively. The variation of (*α* *hν*)^2^ versus *hν* is linear at the absorption edge which confirms that ZrO_2_ is semiconductor with direct band gap. The plot of (*α* *hν*)^2^ versus *hν* is shown in Figure 7. Extrapolating the straight-line portion from higher absorption region of the plot (*α* *hν*)^2^ versus *hν* to photon energy axis for zero absorption coefficient value gives the *E*_*g*_ (Figure 8), giving a value of about 2.49 eV [36]. In the literature, a wide variation has been reported in the band gap of ZrO_2_ which can be attributed to presence of the zirconia phase, defect state, and morphology.

### 3.6. Photoluminescence

Photoluminescence (PL) technique is suitable to determine the crystalline quality and presence of impurities in the materials, as well as exciton fine structure. Figure 8 shows the room temperature photoluminescence spectra of ZrO_2_ NPs excited at four different wavelengths, that is, 270, 290, 300, and 320 nm. Representative fluorescence spectrum with an excitation wavelength of 270 nm shown in Figure 8 exhibited three emission PL bands at around 414, 475, and 563 nm, with 414 nm being the prominent peak. Liang et al. [10] have also reported similar results for t-ZrO_2_. As the fluorescence intensity somewhat varied with the excitation wavelength, the fluorescence band position and the band shape stayed nearly the same for these excitation wavelengths. This indicated that the fluorescence involved the same initial and final states in the excitation wavelengths ranging from 270 to 320 nm. It can be accounted for by fast relaxation from the final state arrived at by photo-excitation to those states from which the fluorescence started [37]. The spectrum features a broad fluorescence band cantered at 414 nm. Although the detailed PL mechanism for the nano-ZrO_2_ is still under research, we could ascribe the emissions that appear at short wavelength excitation to the near band-edge transitions. The PL peak at 414 nm is attributed to Zr vacancies which is one of the intense peaks considered to be due of band edge emission due to the free-exciton recombination [38]. In the case of the emission at 475 and 563 nm, it should be due to the involvement of mid-gap trap states, such as surface defects and oxygen vacancies. The weak green emission also implies that there are surface defects in ZrO_2_ NPs. Large amounts of surface defects exist on the as-synthesized nano-ZrO_2_ particles because of their high surface area. The broad band and the substantial red shift of the band maximum compared to the band gap of the bulk material (5.6 eV) strongly indicate that the fluorescence involves extrinsic states. Because the particle size distribution is very narrow, the broad fluorescence band seems to be mostly caused by the small particle size leading to an inhomogeneous broadening from a distribution of surface or defect states.

## 4. Conclusions

ZrO_2_ NPs have been synthesized with the help of a simple microwave assisted chemical process at low temperature. The TEM analysis reveals that the size of the spherical NPs is in the range of 8–10 nm. All analyses have consistently shown fairly uniform NPs with superfine size having mixed t-ZrO_2_ and m-ZrO_2_ phases. Band gap and PL of the synthesized sample were investigated and band gap of the synthesized ZrO_2_ sample was found to be 2.48 eV from Tauc's relation from UV-Vis absorption spectroscopy. Room temperature PL study of synthesized sample showed three emission PL bands at around 414, 475, and 563 nm, with 414 nm being the prominent peak when excited at 270 nm.
